# Predictors of prolonged mechanical ventilation after heart transplantation

**DOI:** 10.1186/cc11079

**Published:** 2012-03-20

**Authors:** M Turker, P Zeyneloglu, A Pirat, A Sezgin, G Arslan

**Affiliations:** 1 Baskent University, Ankara, Turkey

## Introduction

Several studies have reported that prolonged mechanical ventilation is associated with high mortality and morbidity rates, length of hospital stay, and costs after coronary artery and valvular surgeries. However, no study has focused on the incidence and risk factors of prolonged mechanical ventilation after heart transplantation. The aim of this study was to determine the incidence and predictors of prolonged mechanical ventilation after heart transplantation.

## Methods

We retrospectively analyzed the records of 38 out of 45 patients who underwent heart transplantation from February 2003 to November 2010 at our center. Patients under 12 years of age and those who died before extubation were excluded. We defined prolonged mechanical ventilation as mechanical ventilation longer than 36 hours. Preoperative, intraoperative, and postoperative variables were collected.

## Results

The mean age of the patients (71% male) was 31.5 ± 16.8 years and the incidence of prolonged mechanical ventilation was 40%. Compared with patients who did not require prolonged mechanical ventilation, those who did had significantly lower preoperative hemoglobin levels (12.0 ± 1.5 vs. 13.7 ± 2.4 mg/dl, *P *= 0.03), higher intraoperative lactate levels (7.14 ± 4.13 vs. 3.5 ± 1.82 mmol/l, *P *= 0.006), higher postoperative day 1 serum creatinine levels (2.2 ± 0.9 vs. 1.2 ± 0.7 mg/dl, *P *= 0.002), and longer cardiopulmonary bypass times (143.0 ± 24.2 vs. 122.8 ± 29.1 minutes, *P *= 0.005). Binary logistic regression revealed that the postoperative day 1 serum creatinine level was an independent risk factor for prolonged mechanical ventilation after heart transplantation (OR: 5.109; 95% CI: 1.362 to 19.159, *P *= 0.016). Length of hospital stay was significantly longer in patients with PMV than those who did not require prolonged mechanical ventilation (36.4 ± 30.4 vs. 21.8 ± 12.7, *P *= 0.049). The respective mortality rates for patients with prolonged mechanical ventilation and those without prolonged mechanical ventilation were 60% versus 40%, *P *= 0.15.

## Conclusion

Prolonged mechanical ventilation occurred in 40% of our patients after heart transplantation. A higher creatinine level during the first 24 hours after the surgery was associated with prolonged mechanical ventilation in this study.
